# Experiences with family planning and abortion services during the Covid-19 pandemic: a qualitative study in Bangladesh, Iran and Netherlands

**DOI:** 10.1186/s12889-023-17414-9

**Published:** 2024-01-02

**Authors:** Khadijeh Asadisarvestani, Myrrith Hulsbergen

**Affiliations:** 1 Department of Demography and Geodemography, Faculty of Science, Charles University, Prague, Czechia; 2 Women’s Healthcare Center, Amsterdam, The Netherlands; 3 Teaching & Learning Center, University of Amsterdam, Amsterdam University Medical Center, Amsterdam, The Netherlands; 4 Working Party International Safe Motherhood and Reproductive Health, Amsterdam, The Netherlands; 5 Department of Social Sciences Faculty of Literature and Humanities, University of Sistan and Baluchestan, Zahedan, Iran

**Keywords:** Family planning, Contraception, Abortion, Reproductive health, Sexual health, COVID-19

## Abstract

**Objective:**

Access to family planning services is a human right that plays an essential role in society's health, particularly women’s health. The COVID-19 pandemic has affected all aspects of human life including access to family planning services. Accordingly, the main goal of this study was to explore the experiences of women and service providers from the main challenges and obstacles of access to family planning services and abortion services in Bangladesh, Iran, and the Netherlands during the COVID-19 pandemic.

**Methods:**

In this qualitative study, the data were collected through online, telephone, or in-person semi-structured interviews with key informants. Participants selected by purposive sampling method. The participants included women aged 15 to 49 (*n* = 63) and service providers (*n* = 54) in the 3 abovementioned countries. These individuals were included from October 2020 until December 2020. Conventional thematic analysis was employed to analyze the collected data.

**Results:**

The main extracted themes were challenges (reduction of referral; disruption of access to services; insufficient knowledge; worries among staff; rising prices; and unavailability of some contraceptives), measures (time extension and visit scheduling; telephone, online, and door to door services; and support of the media) and recommendations (health facility improvements; free, online, and closer services; and ongoing trainings and awareness campaigns).

**Conclusion:**

The COVID-19 pandemic has affected family planning and reproductive health services in different ways and has uncovered existing inequalities in access to these services. However, in Iran, the reported challenges were also rooted in new population policies that have further limited access to family planning and abortion services.

## Introduction

An indispensable component of sexual and reproductive health and rights (SRHR) is to enjoy access to family planning and abortion services (FPAs). In 2022, globally, 966 million of 1.9 billion women of reproductive age (15–49 years) were using one or a combination of contraception methods. However, 164 million women wishing to postpone or stop pregnancy permanently were not using any type of contraceptives, showing a high unmet need for contraception worldwide [1]. This unmet need could result in challenges such as unintended pregnancies, unsafe abortion, maternal and infant mortality [2–5].

Emergencies like pandemics could have a negative impact on people’s access to family planning services (FPs) [6, 7] because the main focus of health system is managing critical issues [4, 5, 8]. As an example, a study in Sierra Leone reported that the decline in maternal and newborn care because of suspended services and fear of receiving treatment during the Ebola pandemic resulted in around 3600 maternal and neonatal mortalities and stillbirths, and this rate is close to the number of deaths in this country that were directly caused by the Ebola virus [9].

The COVID-19 pandemic has disrupted FPs [6, 9–13]. For Instance, in Bangladesh, the use of all contraceptive methods except pills plummeted between 30 and 100% right after the lockdown was announced across the nation [13]. The Guttmacher Institute reported that a 10% decrease in sexual and reproductive health services because of COVID-19 could entail an additional 15.4 million unintended pregnancies, more than 3.3 million unsafe abortions, as well as 28 000 maternal deaths [14]. FPAs are essential health services and need to be available during emergencies to save lives and improve quality of life. They can also prevent complications associated with unintended pregnancies, maternal mortality and unexpected increase in sexually transmitted diseases [4, 5, 14]. In countries like Iran and Bangladesh where abortion is legal within a certain period, it is vital to have access to abortion care services without any delay. This is also all the more important because abortion in the first trimester is associated with fewer complications compared with the second trimester [15]. According to one report, 21 477 cases in Iran referred to the legal medicine centers to obtain abortion permission between 2015 and 2017. Of this number, 15 617 (72.71%) cases were approved, but the rest (27.29%) were rejected. Most of these rejections (25.8%) were cases with major anomaly yet their gestational age had exceeded 19 weeks [16].

Evidence supports that access to FPAs around the world is affected by various factors such as fear of COVID-19, economic problems, lockdowns, and suspension of public transportation [17, 18]. However, there are few studies and data for improving FPAs during pandemics. Accordingly, this study by investigating the challenges faced by women and service providers in accessing and providing FPAs can play an effective role in developing interventions during future pandemics and emergencies. Accordingly, the main goal of this international qualitative study was to investigate the barriers and challenges that both service providers (SP) and women of reproductive age faced in the provision of and access to FP and abortion services in 3 countries: Bangladesh (B), Iran (IR), and the Netherlands (NL) during the first 10 months of the COVID-19 pandemic.

### Background of contraceptive use and abortion in Bangladesh, Iran, and the Netherlands

In Bangladesh, based on the latest data, the rate of contraceptive use and unmet need among women of reproductive age is 62.7% (modern methods = 59.1%, traditional methods = 3.6%) and 13.7, respectively [19]. Besides, while it is permitted to do menstrual regulation (MR), abortion is not legally possible except to save a woman’s life. MR is done using manual uterine aspiration or by taking misoprostol (with or without mifepristone) to induce menstruation. This procedure, meanwhile, is legal up to 10 to 12 weeks from the last menstrual period. Although safe and legal MR services are available in Bangladesh, unsafe abortion is a critical issue affecting the health of mothers [20] and it could involve many complications, leaving women in need of post abortion care [21]. In Iran, the rate of contraceptive use and unmet need among women of reproductive age is 77.4 (modern methods = 57.0, traditional methods = 21.7) and 5.7, respectively [19]. Although Iran’s FP is known as one of the most successful programs around the world [22], these rates are from 2010. The population policy has changed from antinataist pronatalist in 2012: in line with the new population policies, the government placed different limitations in accessing contraceptives, which used to be mainly free, and introduced stricter abortion laws. Despite these limitations, women above 40, women with special diseases (eg, cardiovascular diseases and diabetes), and women with children younger than two years of age were considered as high-risk groups and continued to have free access to contraceptives through governmental health centers. Several studies have expressed worries about the consequences of Iran’s new population policies and limited access to FPAs, arguing they would increase the rate of both unintended pregnancies and unsafe abortion and compromise women’s health, particularly for women of low socioeconomic status [23–28]. Nevertheless, since September 2020 the government stopped providing free contraceptives even to high-risk groups. Unsafe and illegal abortions are a major challenge in Iran [29, 30]. Legal abortion is limited to highly deformed or genetically at-risk fetuses; it is also legal for pregnancies with severe complications that reduce the possibility of fetal or maternal survival. Even under these circumstances, abortion is legal before the 19th week of the last menstrual period after the approval of a legal medicine authority ([31] cited by [32]). In the Netherlands, rate of contraceptive use is 73.0 (modern methods = 70.0, traditional methods = 3.0) [19], and its rate of unintended pregnancy and abortion is among the lowest in the world [33]. Furthermore, until the 24th week of pregnancy, abortion can be done without any restrictions. This time limit, however, is not applicable in case of fetal deformity or mother's health risks ([34] cited by [35]).

## Methods

This qualitative study was conducted on 63 women of reproductive age and 54 FPs providers in Bangladesh, Iran, and the Netherlands (Table 1). We attempted to cover different sociocultural groups from urban areas (Bangladesh, Iran, and the Netherlands) semi-urban (Bangladesh and the Netherlands), and rural areas (Bangladesh and Iran). These countries were selected because of their different levels of development, contraceptive use, unmet need rate, and different laws and policies for FPAs. Another reason for selecting these countries was the familiarity of the main researchers with these countries, particularly their FPAs policies as well as culture and language. Convenience sampling was used to recruit qualified participants. We tried to cover different socio-cultural groups aiming to achieve representative sampling, and three different settings including urban, semi-urban and rural areas were selected depending on the country contexts. Eligible women had to be sexually active and between the ages of 15 and 49. However, in Bangladesh and Iran only married women participated in the study because sexual relationship outside marriage is not acceptable. Women under the age of 15 or above 49, participants who did not give verbal or written consent, and women below 16 years in the Netherlands without guardian’s consent were excluded from the study [36].

**Table 1:** Study sample per country

Respondents	Bangladesh	Iran	Netherlands	Total
Women	39	20	4	63
Service providers	28	15	11	54
**Total**	67	35	15	117

Source: [36]

Depending on the context, data were collected using online, telephone, or in-person interview with key informants between October 2020 and December 2020. The interviews (sampling) continued until the saturation point was reached. The semi-structured interviews had 3 main parts: cover letter, questions about socioeconomic and demographic characteristics, and the main questions. The main questions posed to the respondents can be seen in Table 2 [36].

**Table 2:** The interview’s main questions

**Interview the service providers on the provision of contraceptives and abortion services**
Q1: After the lockdown and other prevention measures taken during the COVID-19 situation, have you been able to continue providing FPAs? Please explain the most difficult challenge in providing FPAs during the COVID-19 pandemic
Q2: What measures has your facility taken to ensure that there is ongoing provision of FPAs during the COVID-19 situation?
Q3: In the event of another health emergency in the future, in what ways service provider and regulating bodies both governmental and non-governmental can improve the provision of FPAs?
**Interview the service users on contraceptives and abortion services**
Q1: During the COVID-19 lockdown and after that, have you been able to get the FPAs you needed? Please explain the most difficult challenges to accessing FPAs during the COVID-19 pandemic
Q2: Did you observe any special measures by health care providers to improve your access to FPAs during the COVID-19 situation?
Q3: In the event of another health emergency in the future, in what ways service provider and regulating bodies both governmental and non-governmental can improve the provision of FPAs?

Source: [36]

The original version of the questionnaires was in English and it was translated into 3 languages: Bangladeshi, Persian, and Dutch. Each interview lasted between 45 to 90 min. All interviews were recorded after the appropriate notification had been given to the respondents and their written consent had been acquired. To ensure the quality of the data collection, all interviews were carried out by the principal researchers, who had been involved in protocol and tool development. After all interviews were finalized, the interviews were transcribed and data were analyzed using conventional thematic analysis method. Specifically, the interviews were reviewed several times to gain a full understanding of their content and to identify the primary codes. Then, the codes categorized based on their similarities and differences, and organized under the subcategories. In the next stage, the subcategories were merged under the main categories (Table 3) [36, 37]. In order to increase validity and trustworthiness of the study, we adhered to the analytic procedure that advised by Hseih [37]. In addition, two experts in qualitative research reviewed the data analysis process [37].

**Table 3:** The main extracted themes and sub-themes

Main Themes	Sub-themes
(1) Challenges	1–1. Reduction of referrals
	1–2. Disruption of access to services
	1–3. Rising prices and unavailability of some contraceptives
	1–4. Insufficient knowledge
	1–5. Worries among staff
(2) Measures	2–1-Time extension and visit scheduling
	2–2-Telephone, online, and door-to-door services
	2–3-Support of media
(3) Recommendations	3–1-Health facility improvements
	3–2-Free, online, and closer services
	3–3-Ongoing trainings and awareness campaigns

## Results

In this part, after a brief overview on demographic background of respondents, the main extracted themes will be discussed.

### Demographic background of respondents

A total of 67 qualitative interviews were conducted in Bangladesh in the districts of Dhaka, Cumilla, Cox's Bazar, and Barishal. Among the 39 women interviewed, 13 of them lived in cities and 26 women were in semi-urban and rural areas. Also, 18 women were 21 to 30 years old, and the rest (*n* = 11) were 16 to 20 years of age. Most women (*n* = 15) had secondary school education, and 29 women were homemakers or domestic workers. In Iran, of the 15 service providers, 8 individuals were working in urban areas and 6 in rural areas of three provinces: Fars, Tehran, and Sistan and Baluchestan. All service providers were women. We interviewed 20 women in Iran: 14 lived in urban and 6 in rural areas. Most of respondents in Iran aged between 21 and 30 years, and 3 were below between 18–20 years old. Most of these participants had higher than secondary school education. Besides, 16 women were homemaker. Only 2 women had used abortion services during the COVID-19 pandemic. In the Netherlands, about half of the 11 service providers who were interviewed worked involved in the provision of contraception services, while the rest were in charge of abortion care or referral services. Almost all of these service providers were women. On the other hand, 4 women were interviewed in this country, and all of them had used abortion services during the COVID-19 pandemic. Three of them lived in urban areas and one lived in a semi-urban area. These women aged between 21 and 40 years, and all of them were employed in the Netherlands [36].

### The main extracted themes

Table 3 shows that based on interviews analysis, data can be organized in eleven subthemes placed in three main themes. 1) challenges to accessing FP services (reduction of referrals; disruption of access to services; rising prices and unavailability of some contraceptives; insufficient knowledge; worries among staff); 2) measures taken in clinics or health centers to improve access to FP services (time extension and visit scheduling; telephone, online, and door-to-door services; support of media); and 3) recommendations offered by the participants to promote the quality of and access to FP and abortion services in future crises (health facility improvements; free, online, and closer services; ongoing trainings and awareness campaigns) [36].

### Challenges

The finding of this study revealed that both service users (women) and service providers faced different challenges in accessing and providing family planning services, respectively. These challenges can be divided into five categories as following:

#### Reduction of referrals

The findings revealed a reduction in referrals to FP centers in the 3 countries. According to 2 service providers: “*Consultation time increased due to hygienic measures taken, but challenge was how to get patients to the GP practice; people did not request care” (SP, NL).* [36]. *“Although health centers try to communicate with their clients by phone or online, not all the clients have a telephone line, mobile phone, or internet connection. These are often from the lower socioeconomic classes and are at greater risk of unintended pregnancies” (SP, IR).* [36].

### Disruption of access to services

Due to the implications of the COVID-19 crisis, regulating authorities called for efforts to focus on COVID-19 care, and thereby SRHR services were downplayed. In this regard, we may refer to the following observations: “*The capacity (staff) determines your prioritization” (SP, NL)* [36]. “*At the beginning of lockdown, some of the significant service centers only provided menstrual regulation services on a specific day. But as I am a laborer, it was tough to manage my time and arrange a leave so I could access the services” (W, B)* [36]. However, limitations in access to FP and abortion services were observed more in the first wave of COVID-19:*“Before August during the first wave of COVID-19, it was hard to access FPs. I was pregnant, but Alas! I was unable to make any decision on abortion.” (W, B)* [36]. In Bangladesh, some respondents reported their problem in telephone services: *“Phone counseling was the only option for us to check up or follow up services but it led to an issue as there was only one number dedicated for service seekers (W, B)* [36].

According to the experiences of service providers in the Netherlands, foreign women had more challenges. Furthermore, women across the border could not leave their home country to come to the Dutch clinic or were scared passing due to border checkpoints. Women without adequate health insurance who were unable to leave the Netherlands and return to their home countries due to COVID-19 restrictions had access problems because of financial barriers. Another problem, mentioned by many respondents in Bangladesh, was the disruption of the transportation system. Most of the service providers in Bangladesh experienced long walking distances to reach their clinic, due to lack of transportation and the severe restrictions on moving between neighborhoods areas [36].

It should be mentioned that in Iran, challenges in access to FPs were linked not only to the COVID-19 pandemic but were the termination of free contraceptives for all groups of women, which was mentioned as one of the most important challenges for respondents in Iran:*“Cancellation of free contraceptives has created many challenges, especially for families who cannot afford to pay for contraception. Some women have been forced to use traditional methods due to their inability to obtain contraceptives. This increases the risk of unintended pregnancies. I have seen several unintended pregnancies during this period, and some have been trying to have abortions” (SP, IR)* [36]. *“I know some women who are unable to afford contraceptives. Some of them are reluctant to have intercourse with their husbands for fear of pregnancy, which has caused problems in their relationship with their husbands” (SP, IR)* [36]. *“I used to get the contraceptives for free from the health center, but now it is difficult for me to access contraceptives because there is no pharmacy in our village and I have to use the withdrawal method” (W, IR)* [36].

#### Rising prices and unavailability of some contraceptives

Rising prices of contraceptives, especially condoms, was a problem cited by many respondents in Iran and Bangladesh. Furthermore, the unavailability of some methods made some women both in Iran and Bangladesh step back from modern contraceptives and use the withdrawal method: *“During the COVID-19 pandemic, economic pressure has increased. My husband is a laborer, and if I want to buy a regular condom pack, we have to pay considerable part of our income for it. So I chose to use the withdrawal contraceptive method instead of the condom. It increased my stress about unintended pregnancy” (W, IR)* [36]. *“I used to have cyclofem injection, but during COVID-19 pandemic, [access to] this ampule has decreased and I have to receive medroxyprogesterone injection (quarterly). This change has caused a lot of mental tension for me” (W, IR)* [36].

#### Insufficient knowledge

Lack of sufficient knowledge about sexual and reproductive health issues during the COVID-19 pandemic, especially about pregnancy risks during this period, constituted one such concern mainly among respondents in Iran and Bangladesh:*“I did not intend to become pregnant, but with the onset of COVID-19, I began to feel stressed about what would happen to me and the fetus if I was pregnant” (W, IR)* [36].*“A topic hardly discussed, even on social media, is whether or not we should have intercourse during the COVID-19 pandemic. I have tried not to have sex because I have been afraid of COVID-19 as well as unintended pregnancy” (W, IR)* [36].

#### Worries among staff

Staff shortage was a problem which created pressure on the existing health care staff in Bangladesh: ***“*** *There was not enough human resources or not even enough service providers in the clinics to provide service” (SP, B).* Furthermore, service providers in both Bangladesh and the Netherlands reported difficulties in organizational and safety tasks and a lack of management guidance and supervision on the provision of services. Partly due to insufficient resources, implementing personal protection equipment (PPE) protocols was difficult, causing a reduction in service provision and the risk of contracting COVID-19 at work:

***“*** *At the beginning of COVID-19, it was tough to provide services as there was not enough health measurement. As an example, there was only 1 PPE for 10 doctors, and they had to exchange it among themselves and so breach the COVID-19 protocols” (SP, B)* [36]. *“There are other GPs who are very reluctant (to see patients). And that is partly due to personal fear, like how do you estimate the risk. We may be a little more light-hearted (in our practice) about this” (SP, NL)* [36]. Furthermore, some staff were worried because some clients would ignore the health protocols by some client: “*Sometimes we could not keep physical distance as service seekers were not aware of the pandemic or were not enough concerned about maintaining health measures of sanitization or using masks and other protection equipment*” (*SP, B)* [36].

### Measures

Despite challenges during the COVID-19 pandemic, measures to family planning services were taken by the service providers to improve access to FPs in the 3 countries and to abortion services in Bangladesh and the Netherlands [36]. These measures can be divided into three categories that will be discussed in the following section.

#### Time extension and visit scheduling

The extension of service time was a strategy employed in the 3 countries. Scheduling client visits was another strategy in Iran and Bangladesh to increase access to FPs in a safe environment: “*I had to go for injection. So I called the health center and they appointed a time for me. Then on that specific day, I received the FP service without any hesitation” (W, B)*; “*Most of the pharmacies were open during lockdown so menstrual regulation kits were available to help women avoid unintended pregnancies” (W, B)* [36].

#### Telephone, online, and door-to-door services

Telephone and online consultations and working from home were among measures which were taken by some service provides in the 3 countries: ***“*** *In fact, this is a national guideline, and we provide free counseling during office hours to people who need counseling services concerning contraceptive use and abortion” (SP, IR* [36]. *“Once the condom broke during intercourse, and I was very worried about getting pregnant. Upon my phone call to the health center, they gave complete instructions on emergency contraception and, fortunately, no pregnancy occurred” (W, IR)* [36].

Although the delivery of online services by health centers was not common in Iran before COVID-19 pandemic, some of them attempted to provide online advice during the pandemic because of the close relationship of the staff with clients: *“I have been working in the health center of this village for more than 20 years, and I have family relationship with some clients. Indeed, I know some of these women, for example, the less educated women or women who are in high-risk groups in terms of pregnancy or those who may face more problems. So during the COVID, I would contact them *via* WhatsApp or Telegram and give them the necessary advice” (SP, IR)* [36].

In Bangladesh, the clinic authorities encouraged door-to-door services. Some respondents said that in some rural areas, group services and counseling were offered by service providers so that women did not have to travel to the clinic: *“In some places, zone wise duties have been introduced for overcoming the distance problem of service providers. Even after July 2020, door-to-door service was introduced to improve FP access for women who are not able to receive services [in the usual manner]” (SP, B.)* [36].

In the Netherlands, one of the measures taken by the Women on Web (WoW) service was an appeal to the government through the court to allow online abortion clinic counseling and the prescription of abortion pills by a general practitioner (GP). One woman, living in a semi-urban area, pointed out that she would have had problems accessing an abortion clinic if her GP had not helped her by ordering abortion pills for her. In Iran, the situation of abortion services was different: almost all the SPs (90.0%) reported that no change was made in the provision of abortion services during the COVID-19 pandemic [36].

#### Support of media

The Netherlands and Bangladesh made adjustments in (social) media, including the use of a national newsletter for GPs to emphasize the continuation of contraception and abortion care and the need to raise clients’ awareness. Onlin adjustments were applied both to inform the public about the continuation of services and COVID-19 measures and to triage patients. Preparing roadmaps, organizing meetings, and prioritizing acute care services were emphasized in this context [36].

### Recommendations

One of the main parts of this study was to find out the respondents' views on ways to improve family planning services, especially in times of crisis. The recommendations of respondents can divided into three main categories:

#### Health facility improvements

Some service providers in the Netherlands recommended that a handbook about contraception, abortion, and menstrual regulation as well as task division between different service providers and crisis management should be available during crises. In addition, some service providers in Bangladesh advised collaboration between the government and non-government organizations to avoid replication of programs and services. Also, to improve access to services, they recommended that flexible service hours be adopted and even the opening hours of the centers increase when client demand is high. In case of extra service hours, they highlighted that more service providers should be recruited quickly [36].

Several respondents in the 3 countries emphasized better communication and friendly disposition of service providers. They mentioned that such communication with clients will encourage a wider range of people to visit health centers and receive the necessary services. Consequently, health centers will better monitor the population under coverage in all situations, especially in emergencies. One woman stated,* “Service providers should be well trained so that a comfortable and friendly environment could be ensured for women” (W, B).* Furthermore, since some women and service providers in Bangladesh had transportation problems, they believed improvement of the transportation system is necessary to ensure the availability of service providers at clinics and women’s access to FPs [36].

#### Free, online, and closer services

Some service providers recommended the provision of free or affordable contraceptives for all women, particularly for migrants, and the inclusion of contraception in the basic health insurance. Most respondents in Bangladesh recommended that online counselling should be in place to be able to give follow-up care to women at home. Several respondents called for the continued provision of online and telephone consultations after COVID-19: *“Free counseling online and offline should be dedicated for all married and single women, especially those who are more vulnerable and marginalized”* (W, B*).* The need to restart free access to FPs was frequently raised by respondents in Iran: ***“*** *It is better to have contraceptives available, as in the past, free of charge to all people, especially for couples who are less literate or come from lower economic classes. Besides, it should be borne in mind that in times of crisis, such as the present, some people face economic problems. Hence, they may refuse to purchase a contraceptive. Thus, it is better, at least in these circumstances, that the device be available to everyone for free” (SP, I)* [36].

Most of the responding women in Bangladesh urged improving the accessibility of door-to-door services or one-stop service for women who are unable to leave the house: *“Some women are unable to leave home in times of crisis because of a specific disease, their small child, a sick family member, and the like. Therefore, it is best to identify such people and provide them with contraceptives at their place” (W, B)* [36].

#### Ongoing trainings and awareness campaigns

Education about FP and reproductive health and how to cope with problems in times of crisis should be one of the major educational agendas of the ministry of health of a country: *“Now that fertility growth policies have been adopted in Iran, FP education should not be underestimated or neglected. Both before and during the crisis, people should receive education regarding contraceptives, reproductive health in times of crisis such as the COVID-19 outbreak, pregnancy risks, pregnancy preservation, and more” (SP, I)* [36].

In addition, media can be deployed to improve people’s awareness: *“Official media can play an important role in times of crisis. Unfortunately, FP issues have not received much attention from the national media, which can provide the necessary training and information” (SP, I).* In the 3 countries, the online and offline approaches of knowledge sharing platforms can be combined to improve people’s knowledge about and access to FPs. Information should be delivered in easy and clear language. There should be a balance between digital and traditional modes of knowledge sharing and education on contraception and abortion. Furthermore, some respondents in the 3 countries stressed the need to arrange outreach campaigns and meetings, including more male members to create awareness and highlight the importance of contraceptive provision, reproductive health, and FP [36].

## Discussion

The findings of this study revealed that similar to many countries, the provision of family planning and abortion services was affected by COVID-19 to varying degrees in Bangladesh, Iran, and the Netherlands [35, 36, 38–40]. The most notable barriers and challenges in all these countries were the reduced client flow, staff shortage, and change in opening hours. Cost of contraceptives and the shortage of supply were challenges both in Bangladesh and Iran. Also, shortage of personal protective equipment was one of the main challenges in the Netherlands and Bangladesh, which augmented service providers’ concern regarding their own health and that of clients [36].

In line with previous studies, we found that immigrant women and women from lower socioeconomic categories and rural areas experienced greater inequalities in access to family planning services prior to the COVID-19 pandemic [36, 41–43]. Likewise, a study found that during COVID-19 pandemic in Bangladesh, a woman from rural areas was about 65% less likely to receive FPs than someone living in urban areas [44]. Another study in the Netherlands also suggested that women residing in places without abortion clinics faced more access barriers to abortion services [45]. Meanwhile, in the case of Bangladesh, other studies have noted that even before the COVID-19 pandemic, rural women had a lower chance of receiving contraception and MR services than urban women [24, 36, 46].

Despite some similarities between the 3 countries, Iran had some differences both in terms of barriers and strategies. Regarding barriers, access to contraceptives in Iran was affected not only by the COVID-19 but also the new population policies in the country. This situation created problems particularly for women who were living in rural areas and women from lower socioeconomic classes. Furthermore, despite all challenges caused by the COVID-19 pandemic, family planning service providers particularly in Bangladesh and the Netherlands employed different strategies (such as online and phone consultation or rotational duties, and providing information for service users via media and adjusting service hours or work-plans) to improve access to services. In Bangladesh, users were offered more accessible, door-to door family planning and abortion services. In the Netherlands, the so-called WoW initiative tried to get the legal permission to allow online abortion clinic consultations and the prescription of abortion pills by GPs. In Iran, strategies were mainly limited to providing consultation (online, by phone, and in-person) but not contraception devices through public health centers. In terms of abortion, as discussed earlier, the abortion law in Iran is strict and the government did not implement any special measures to improve access to legal abortion [36, 47]. The media in both Bangladesh and the Netherlands attempted to improve access to family planning and abortion services during the COVID-19, but in Iran did not see any media activity in this regard. As a result, inadequate knowledge about family planning and reproductive health issues was disconcerting for Iranian women during the COVID-19 pandemic [36].

Certain recommendations for policymakers could be extracted from the results of this study:

- ▪ Government awareness initiatives and (online) knowledge development programs addressing sexual and reproductive health at times of emergency (all 3 countries) [36].
- ▪ Offering free or inexpensive family planning services (all 3 countries) [36].
- ▪ Ensuring free and safe transportation services for emergency service providers in future crises (Bangladesh) [36].
- ▪ Addressing the critical role of family planning and sexual and reproductive health in the society’s health and as a basic human right in population policies (Iran) [36].
- ▪ Continuity of services through various means such as offering services by online/telephone counseling (all 3 countries) [36].
- ▪ Taking family planning services closer to the user and promoting door-to-door contraception services (Iran and Bangladesh) [36].
- ▪ Improving health facilities by developing a handbook for health care staff on sexual and reproductive health under emergency circumstances (the Netherlands), promoting collaboration of health care staff, adjusting opening hours, and providing adequate and efficient supplies (Bangladesh) [36].
- ▪ Amending restrictive abortion laws at times of a pandemic to mitigate the rate of unsafe abortion and their complications (Iran and Bangladesh) [36].

### Strengths and limitations of the study

This study was an international study which was conducted in three countries with different levels of development and structures of family planning services. Furthermore, the method of this study was qualitative; as a result, we gained in-depth knowledge about accessing challenges to family planning services. However, this study has some limitations. The population of this study was limited to women. Besides, as this qualitative study was conducted on a small group of women in three countries, its findings may be limited in terms of generalization.

## Conclusion

The COVID-19 pandemic impacted access to family planning and abortion services in different ways, highlighting inequalities in access to these services. The findings also demonstrated that immigrant women, rural women, and those from less economically and socially privileged groups faced more challenges in accessing family planning services. However, provision and access to family planning services in the Netherlands were less affected by COVID-19 compared to Bangladesh and Iran. In the case of Iran, both barriers and strategies in relation to providing sexual and reproductive health services have also been affected by the introduction of pronatalist policies and regulations that restrict access to family planning and abortion services [36].

## Data Availability

The datasets generated and/or analyzed during the current study are not publicly available due to ethical reasons and to protect the integrity of participants, but are available from the corresponding author on reasonable request.
